# Decadelong low basal ganglia NAA/tCr from elevated tCr supports ATP depletion from mitochondrial dysfunction and neuroinflammation in Gulf War illness

**DOI:** 10.1038/s41598-025-24099-0

**Published:** 2025-11-20

**Authors:** Sergey Cheshkov, Lisa C. Krishnamurthy, Audrey Chang, Hyeon-Man Baek, Sandeep Ganji, Evelyn Babcock, Jeffrey S. Spence, Richard W. Briggs, Robert W. Haley

**Affiliations:** 1 Department of Radiology, University of Texas Southwestern Medical Center, 5323 Harry Hines Blvd., Dallas, TX 75390-9113 USA; 2 Advanced Imaging Research Center (AIRC), University of Texas Southwestern Medical Center, 2201 Inwood Road, Dallas, TX 75390-8568 USA; 3 Department of Clinical Sciences, University of Texas Southwestern Medical Center, 5323 Harry Hines Blvd., Dallas, TX 75390-8874 USA; 4 Division of Epidemiology, Department of Internal Medicine, University of Texas Southwestern Medical Center, 5323 Harry Hines Blvd., Dallas, TX 75390-8874 USA; 5 Present Address: Center for BrainHealth, University of Texas at Dallas, 2200 W. Mockingbird Lane, Dallas, TX 75235-5451 USA; 6 Present Address: Joseph Maxwell Cleland Atlanta VA Medical Center, 1670 Clairmont Road, Decatur, GA 30033 USA; 7 Present Address: Department of Radiology and Imaging Sciences, Emory University School of Medicine, 1364 Clifton Rd N E, Atlanta, GA 30322 USA; 8 Present Address: Center for Translational Research in Neuroimaging and Data Science, 55 Park Place NE, Atlanta, GA 30303 USA; 9 Present Address: Lee Gil Ya Cancer and Diabetes Institute, Gachon University School of Medicine, 191 Hambakmoe-ro, Yeonsu-gu, Incheon Metropolitan City, 21936 South Korea; 10 Present Address: Philips, Cambridge, MA USA; 11 Present Address: Department of Radiology, Mayo Clinic College of Medicine, Rochester, MN USA; 12 Present Address: Epidemiology Unit, Division of Infectious Diseases and Geographic Medicine, Department of Internal Medicine, and Epidemiology Department, O’Donnell School of Public Health, University of Texas Southwestern Medical Center at Dallas, 5323 Harry Hines Boulevard, Dallas, TX 75390-9113 USA

**Keywords:** Mitochondrial diseases, Neuroinflammatory diseases, Proton magnetic resonance spectroscopy, Veterans, Persian Gulf syndrome, Computational biology and bioinformatics, Molecular biology, Environmental sciences, Molecular medicine, Pathogenesis

## Abstract

Reduced N-acetylaspartate (NAA)/total creatine (tCr) ratio found with long echo-time proton magnetic resonance imaging (^1^H-MRS) of deep brain structures in a Seabees Battalion in 1997–1998 was soon replicated by two studies but not in a later one using intermediate echo time. We undertook this study in 2008–2009 to repeat the previous long echo-time ^1^H-MRS study with ^1^H-MRS at both long (TE = 270 ms) and short (TE = 30 ms) echo time and higher field strength (3T) to test whether the abnormality of NAA/tCr affecting this Battalion had normalized or been obscured by effects of the T_2_ decay curve. Under investigator blinding, 39 Seabees in the three GWI variant groups and 16 Seabees controls prospectively underwent ^1^H-MRS at both short (TE = 30 ms) and long (TE = 270 ms) echo time to measure metabolites and at five TE values between 60 ms and 270 ms to measure transverse relaxation time (T_2_) in the basal ganglia. A mixed-effects linear model adjusting for age tested group differences. Findings supported the observations of the prior studies, demonstrating that veterans with GWI no longer had reduced NAA/tCr at long echo time but had significantly lower basal ganglia NAA/tCr than controls at short echo time (left: 1.22 ± 0.02 vs. 1.38 ± 0.03, *P* < 0.0001; right: 1.12 ± 0.02 vs. 1.18 ± 0.03, *P* = 0.059). The group differences were mainly due to higher [tCr] (left: 14.1%, *P* = 0.0001; right: 9.1%, *P* = 0.009) rather than lower [NAA] in the ill groups. Longer echo time substantially reduced the sensitivity of ^1^H-MRS. Chronic metabolite abnormalities persisted in GWI for 10 more years but remained detectable only at short echo time. That reduced NAA/tCr is due primarily to increased [tCr] rather than decreased [NAA] supports recent studies implicating mitochondrial dysfunction with ATP depletion and neuroinflammation as causative factors and therapeutic targets in GWI.

**Supplementary Information:**

The online version contains supplementary material available at 10.1038/s41598-025-24099-0.

## Introduction

Gulf War illness (GWI), affecting over 25% of the 700,000 military personnel deployed to the 1991 Persian Gulf War^1^, features a variety of symptoms including fatigue, pain, memory/concentration problems, balance disturbances, post-exertion malaise, chronic diarrhea, skin rashes and depression. Studies of a gene-environment interaction strongly support the causative role of low-level sarin nerve gas exposure from U.S. and Coalition bombing of Iraqi chemical weapon production and storage facilities^2,3^. In 2012 Golomb first suggested mitochondrial dysfunction as the underlying mechanism of GWI based on overlap of their symptoms^4^. In three subsequent studies she and colleagues supported the hypothesis with phosphorus magnetic resonance spectroscopy (^31^P-MRS) demonstrating prolonged recovery time of muscle phosphocreatine after exercise, a direct measure of mitochondrial function, in veterans with GWI^5–7^ and with response of some GWI symptoms to treatment with co-enzyme Q10, a known treatment for mitochondrial dysfunction^8^. Concurrently, O’Callaghan and colleagues described with a preclinical rodent model that repetitive low-level doses of the sarin proxy diisopropylfluorophosphate (DFP) produces a lasting behavioral disturbance in rodents related to neuroinflammation^9–11^ possibly from mitochondrial dysfunction^12^. Deshpande and colleagues reported increased intracellular calcium concentration in a similar GWI rodent model^13,14^. Human studies suggest that these pathological processes are active in Gulf War veterans also^5,7,15^.

Among the early mechanistic studies of GWI was the report in 2000 of the first proton magnetic resonance spectroscopy (^1^H-MRS) brain scans of GWI cases and controls performed in 1997–1998^16^, the third in a longitudinal series of epidemiologic and clinical studies to understand GWI in the 24th Reserve Naval Mobile Construction Battalion (Seabees)^16–18^. That study applied long echo-time (TE = 272 ms) ^1^H-MRS at 1.5 Tesla (T) field strength in 22 GWI cases and 18 well veteran controls drawn from prior studies. It showed a statistically significant reduction of the N-acetylaspartate/total creatine ratio (NAA/tCr) in the right basal ganglia and pons, thought at the time to suggest neuronal damage.

A year later, Meyerhoff et al. replicated the finding by demonstrating reduced NAA/tCr and NAA/choline ratios in right basal ganglia with long echo time (TE = 270 ms) ^1^H-MRS at 1.5T in 11 GWI veterans and 11 controls selected from a VA Gulf War clinic^19^. In 2004 the first application of short echo time (TE = 30 ms) ^1^H-MRS at 1.5T, Menon et al. found reduced NAA/tCr in the hippocampi of GWI veterans bilaterally^20^. In 2011 Weiner et al. published a larger study using intermediate echo-time (TE = 135) at 4T in Gulf War veterans recruited through public advertising found no difference from controls in [NAA], NAA/tCr and NAA/Cho in basal ganglia, pons, hippocampus, and gray and white matter, suggesting that the previously demonstrated abnormality might have normalized over time^21^.

A decade later the longitudinal sample was enlarged with additional GWI veterans from the Seabees Battalion for a fourth study in the longitudinal series. We designed the restudy to test Weiner’s hypothesis by repeating the ^1^H-MRS at the same long echo time (TE = 270 ms), expecting normalization, and adding a more powerful short-echo-time scan (TE = 30 ms) to detect possible weaker signal of residual disease in the chronic phase. As before, the protocol included scanning a single voxel in the basal ganglia bilaterally and analyzing the concentrations of N-acetylaspartate (NAA), total creatine (tCr) and the NAA/tCr ratio, only this time at 3T field strength instead of 1.5T to obtain more precise estimates of metabolite concentrations.

## Methods

### Subjects/groups

**Fig. 1:**
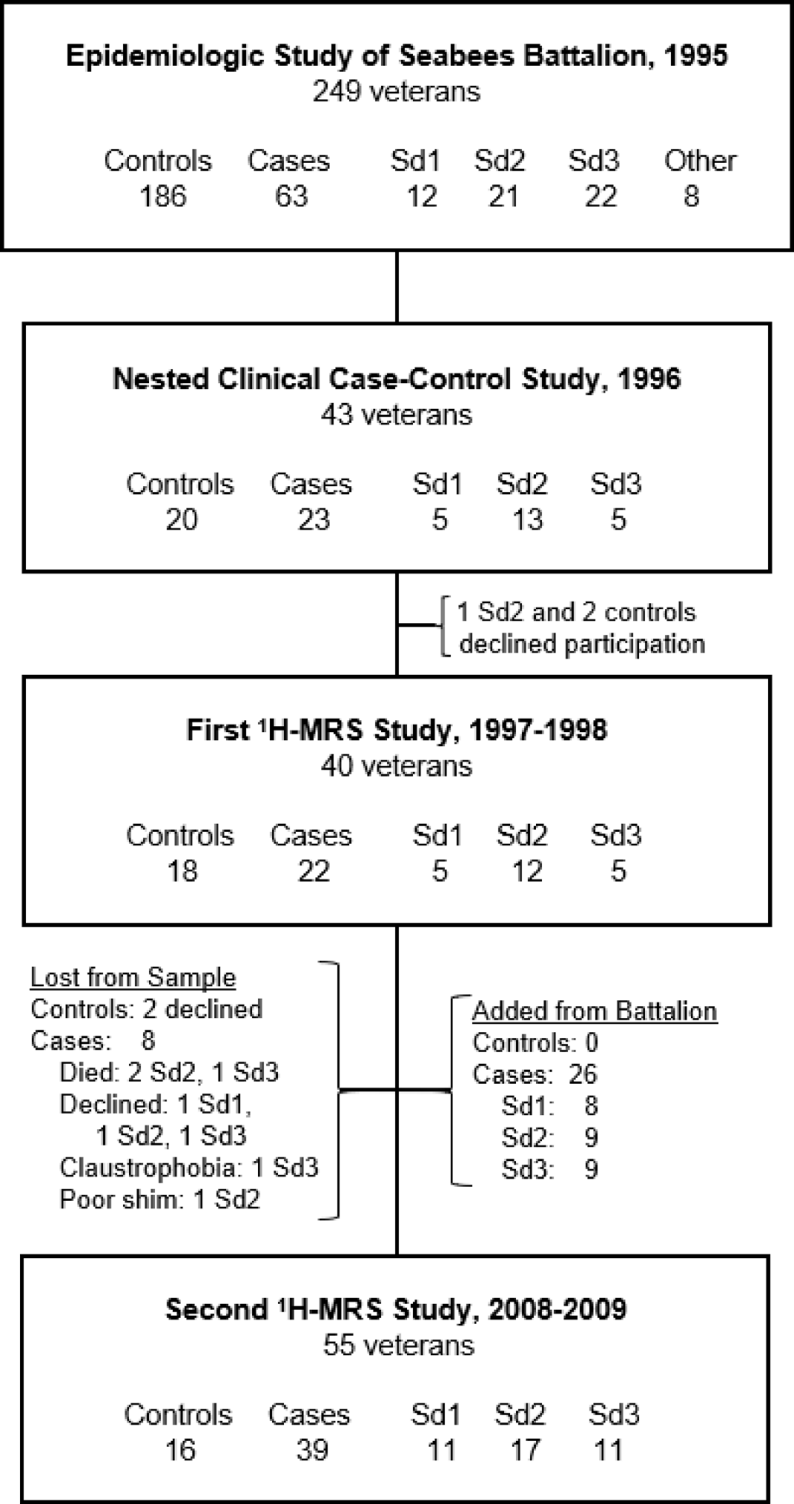
Flow chart of the Gulf War veteran samples of the Seabees Battalion across the 4 studies of the 23-year longitudinal study.

For inclusion in these studies, veterans must have served in the Seabees Battalion during the 1991 Gulf War, and GWI veterans had to meet the original GWI Research case definition^17^, a subset of the later CDC and Kansas definitions^22,23^, that subclassifies ill veterans into 3 GWI variants: syndromes 1 (impaired cognition), 2 (confusion-ataxia) and 3 (neuropathic pain). Seven of the 16 Seabees controls were not deployed to the war zone. The only exclusion criteria were those that precluded MRI. None served in military actions after the 1991 Gulf War. This study was the fourth in a 23-year longitudinal study of GWI in this battalion (Fig. 1). Over the 10 years between the third and fourth studies, the sample lost eight participants, and claustrophobia and a poor shim excluded two more. Twenty-six additional Battalion members were added to ensure adequate statistical power (Fig. 1). All were included in a national study of 8,021 Gulf War veterans^24^. Demographic and clinical characteristics of the 55 education-matched right-handed cases and controls are given in Table 1. The groups were comparable on all measures except age which we therefore controlled for in the main analysis of group effects.

**Table 1:** Demographics and clinical information for seabees subjects from the 24th Reserve Naval Mobile Construction (Seabees) Battalion. *Of the controls, 9 were deployed and 7 were non-deployed, all from the same seabees battalion. † P values for age and education were obtained from an F-test with 3 df, and those for the rest, from Fisher’s exact test for a 4 × 2 table. ‡ no information was provided by one deployed control subject.

Characteristic	Controls*	Syndrome 1	Syndrome 2	Syndrome 3	*P* value†
N of subjects	16	11	17	11	
Mean age, years (SD)	60.4 (6.8)	51.2 (6.1)	62.7 (6.7)	57.3 (6.5)	0.0003
Mean years of education (SD)	13 (2)	14 (1)	12 (3)	12 (1)	0.09
Alcohol abuse or dependence (%)	4/15 (27)‡	4/11 (36)	6/17 (35)	5/11 (45)	0.80
Drug abuse or dependence (%)	2/16 (13)	1/11 (9)	1/17 (6)	2/11 (18)	0.80
Major depressive disorder (%)	0/15 (0)‡	2/11 (18)	3/17 (18)	0/11 (0)	0.16
Post-traumatic stress disorder (%)	0/16 (0)	2/11 (18)	5/17 (29)	2/11 (18)	0.11

The study was conducted according to the Declaration of Helsinki and the Belmont Report, and all subjects gave written informed consent for the study protocol, approved by the Institutional Review Board of the University of Texas Southwestern Medical Center, before the study procedures began.

### Data collection

**Fig. 2:**
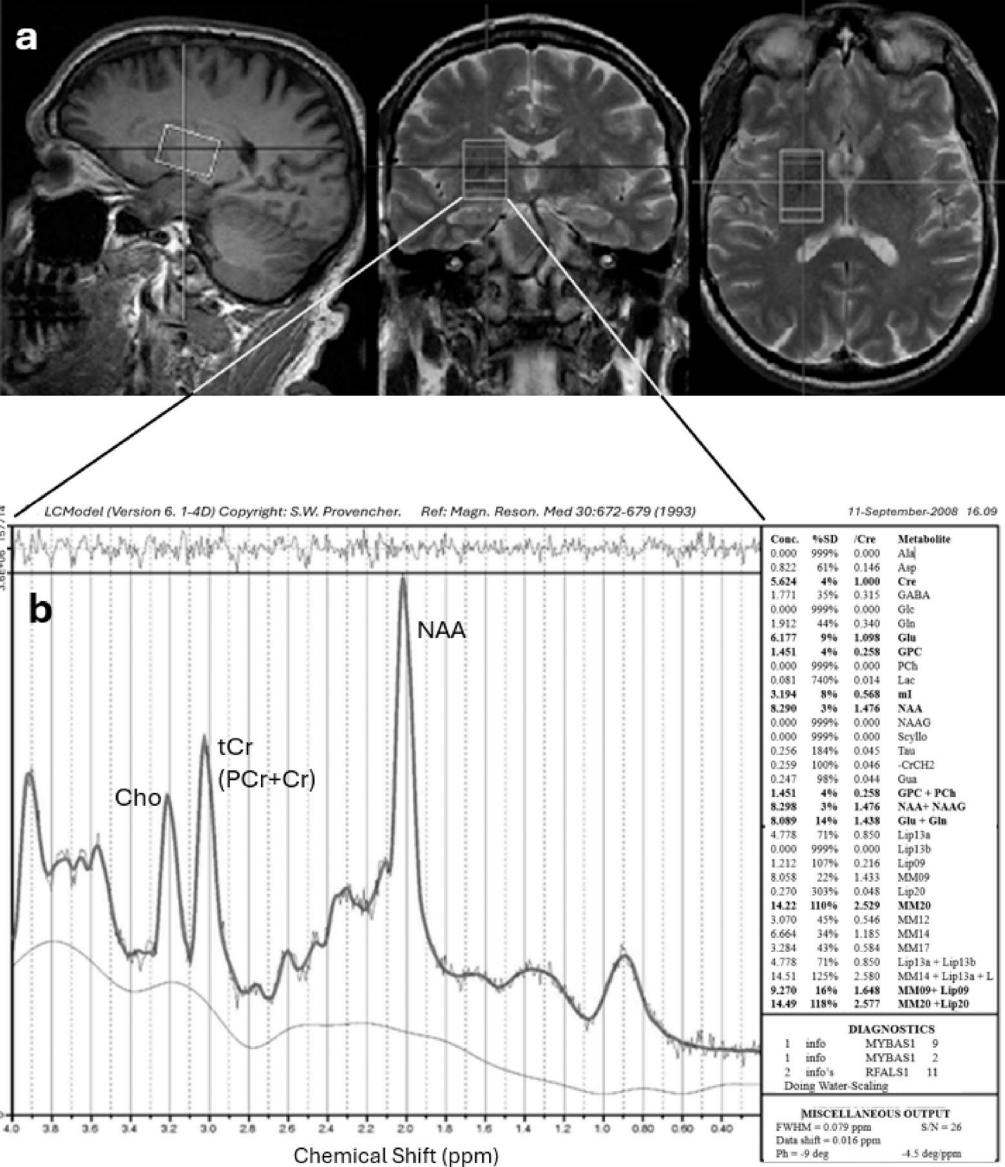
Acquisition of spectra from a Gulf War veteran with LCModel to estimate concentrations of N-acetylaspartate (NAA), total creatine (tCr) and choline (Cho). (**A**) Spectroscopy voxel positioning in the right basal ganglia. (**B**) A 3T ^1^H-MR spectrum (TE = 30 ms) of the left basal ganglia of a representative subject processed using LCModel. Metabolites detected with acceptable reliability are shown in bold font in the table on the right.

Localized ^1^H-MRS data were collected using a single-voxel point-resolved spectroscopy sequence (PRESS) on a Siemens 3T Trio total imaging matrix (TIM) MR system with a 12-channel head coil and TR = 2500 ms, TE = 30 ms (96 averages, acquisition time = 4:10) and 270 ms (64 averages, acquisition time = 2:47). Single voxels were centered in the putamen bilaterally, catching small corners of the caudate head and anterior thalamus, and excluding ventricular cerebrospinal fluid [CSF] with volume = 12.0 mL (20 mm x 30 mm x 20 mm, LR/AP/IS) (Fig. 2A). It had spectral width = 2000 Hz, water suppression bandwidth = 50 Hz, data points = 1024. An unsuppressed water spectrum with eight averages was acquired for eddy current compensation^25^ and quantitation.

High resolution localizer images in 3 orthogonal planes were used in combination with Siemens AutoAlign to ensure accurate and reproducible voxel positioning among subjects and across multiple scan sessions within subjects and avoiding white matter and ventricles to prevent confounding by tissue type^26^ (Fig. 2A). Magnetic field homogeneity was achieved by standard auto-shim followed by first order manual shimming.

For determination of metabolite T_2_ relaxation times, data were collected for each subject with TR = 2500 ms at five echo times (TE = 60 ms, 90 ms, 135 ms, 195 ms, 270 ms), with 32 averages at TE ≤ 135 ms and 64 averages at TE ≥ 195 ms to compensate for decreasing SNR at higher TE values. TE = 30 ms data were not used in the T_2_ calculations because of the contamination of the 3.0 ppm tCr peak with macromolecule resonance at that TE value, which was not accounted for in the jMRUI fit used for the T_2_ data analysis. The TE = 270 ms data, with 64 averages due to time constraints from the suite of TE values used for T_2_ calculations, was also used for metabolite quantification to compare to metabolite quantifications obtained from 96 averages at TE = 30 ms.

Coefficients of variation of the data produced by these methods have been demonstrated in reproducibility studies of normal controls to be about 3%, 5%, and 7% for [tCho], [tCr], and [NAA] quantification, respectively; <8% for [NAA] T_2_ measurement; and < 17% for [tCr] and [tCho] T_2_ measurements^27,28^.

### Spectral processing

The TE = 30 ms and TE = 270 ms ^1^H-MRS data were processed for metabolite quantification with LCModel^29,30^ using the 3T Siemens basis set of 26 metabolites including lipid and macromolecule signals, obtained with the software. A basis set including glutathione was derived by theoretical simulation of metabolite spectra with VESPA (http://scion.duhs.duke.edu/vespa/project).

Analysis of data collected for determination of T_2_ consisted of Hankel-Lanczos singular values decomposition (HLSVD) filtering of residual water signal, apodization of 5 Hz, phasing, and quantification of [NAA], [tCr], and [tCho] using the non-linear AMARES algorithm of the jMRUI software package^31^. T_2_ values were calculated from least-squares fits of the logarithm of the peak areas vs. TE values after correction for the different number of averages used at the various TE values. The T2 decay curves were modeled as bi-exponentials, and a single exponential decay was found to give the best result, consistent with rapid exchange averaging.

To correct for partial T_1_ saturation at longer TE values in the constant TR paradigm, which if not accounted for leads to systematic underestimation of T_2_ values^32,33^, the intensity at each TE value was corrected by dividing by the following correction factor^33^:

f(T_1_,TR,TE) = 1–2exp[-(TR-3TE/4)/T_1_] + 2exp[-(TR-TE/4)/T_1_] - exp[-TR/T_1_].

For TR = 2500 ms, using for T_1_ the longest (1.55 s) of the metabolite T_1_ values measured at 2T and 3T from the literature^34–36^, this correction factor differs by 3% between the shortest and longest TE values used in this study.

### Statistical analysis

We designed the study to scan members of the same Seabees battalion with both long- and short-echo-time ^1^H-MRS to test the specific hypothesis, raised by the 4 prior studies, that the inability to replicate the initial reduced NAA/tCr ratio^18–20^ 10 years later^21^ was due to its signal fading over time. We modeled each metabolite and ratio using a mixed-effects linear model.

$${y_{{\text{ijk}}}}\,=\,{\mu_{{\text{ij}}}}\,+\,{x_{\text{k}}}\,+\,{\epsilon_{{\text{ijk}}}},$$

where *y*_ijk_ is the metabolite concentration (or the ratio) for the j^th^ hemisphere of the k^th^ subject in the i^th^ group; *µ*_ij_ is the mean of the i^th^ group and j^th^ hemisphere, *x*_k_ is the age of the k^th^ subject; and *ε*_ijk_ is a random error term for which subjects are independent but hemispheres within subject are correlated. We used a variance component model as the covariance structure of *ε*_ijk_, a 2 × 2 matrix for each of the k subjects, where the off-diagonal elements are the between-subject variance component and the diagonal elements are the sum of the between- and within-subject variance components. Group-level contrasts of means were of the form *µ*_ij_ - *µ*_i’j_ within each hemisphere, specifically for ill groups against the control group. We included age in the linear mixed model to control for its possible effect on metabolites or their ratio due to the fact that mean ages were significantly different across the groups (F_3,51_ = 7.25, *p* = 0.0004; Table 1), syndrome 1 having the lowest. Each omnibus ANOVA test used a 4-degree-of-freedom F statistic, and each contrast, a t statistic. We used the two-stage linear step-up procedure described by Benjamini et al.^37^ to protect the false discovery rate (FDR) over the set of all contrasts and metabolites within each of the short- and long-echo-time data at the α = 0.05 level. The association of GWI with the absolute myo-inositol concentration was tested in the right and left basal ganglia with 4-group ANOVAs with 2-group contrasts testing the association of each of the 3 syndrome groups with the control group. In a third analysis with both sides pooled, the association was tested with a linear mixed model including contrasts accounting for the correlation between the sides. Initially significant P values of the 2-group tests remained significant if the familywise error rate was ≤ 0.05 by Dunnett’s procedure.

## Results

### Data quality

Metabolite half-height line widths were 9–14 Hz. Cramer-Rao lower bounds were 3–4% for [tCr], [NAA], and [tCho] fits. Less than 3% of data sets for T_2_ calculations were rejected due to poor fit quality. A typical example of a spectrum is shown in Fig. 2B.

### Control groups

Since there were no significant differences between the deployed and non-deployed control groups in metabolite concentrations or ratios (*P* > 0.25) or T_2_ values (*P* > 0.40) of the three major metabolites (NAA, tCr, tCho), data for the two control groups were pooled for further analyses.

### Metabolite concentrations and ratios

**Fig. 3:**
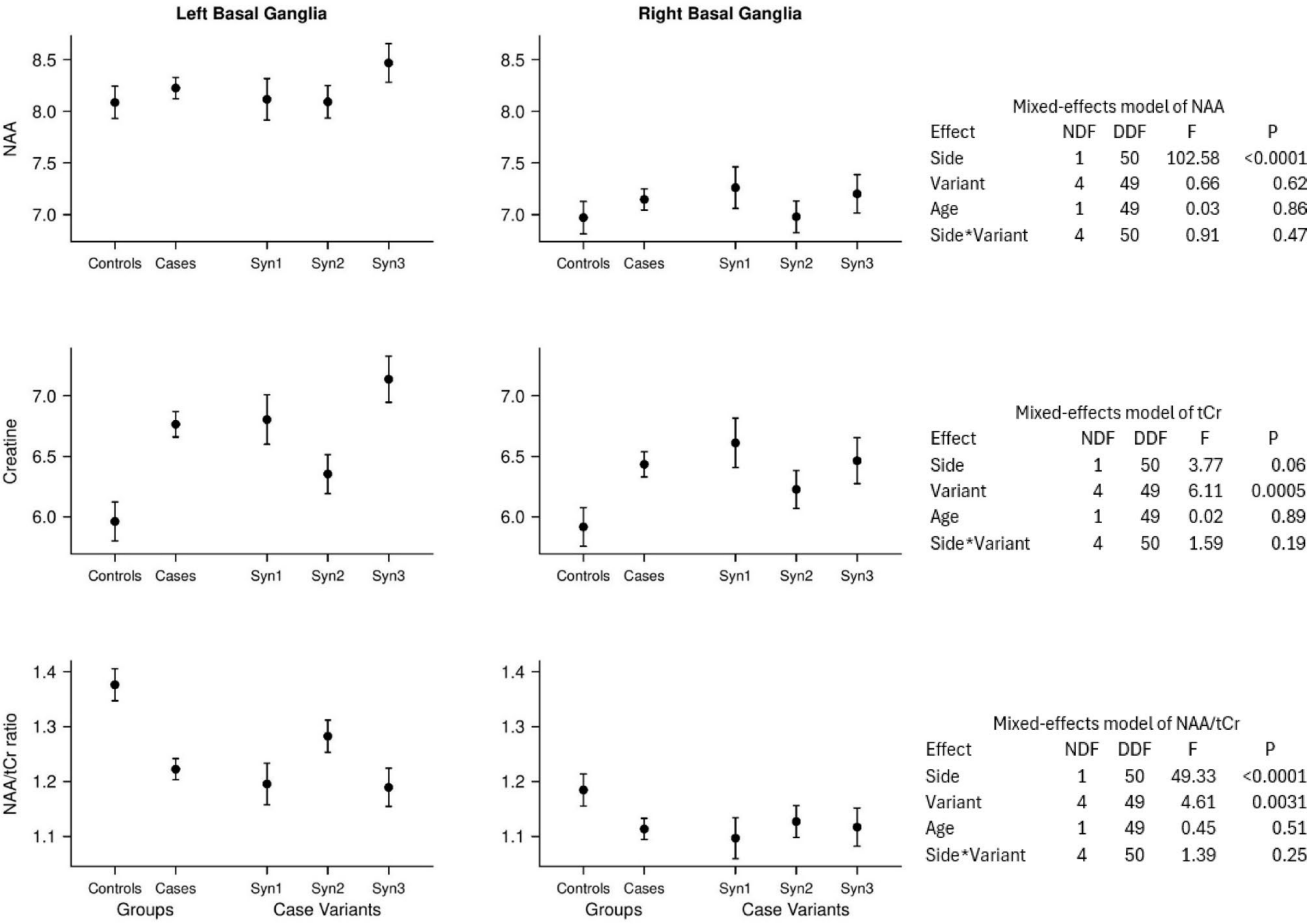
Mixed-effects linear models of [NAA] (N-acetylaspartate), [tCr] (total creatine) and their ratio (NAA/tCr) measured at short echo time (TE = 30 ms). For each outcome, the model tested for differences between the left and right basal ganglia (Hemisphere), between controls and case variants (Groups), and the Hemisphere-by-Group interaction. The model also controlled for age.

**Fig. 4:**
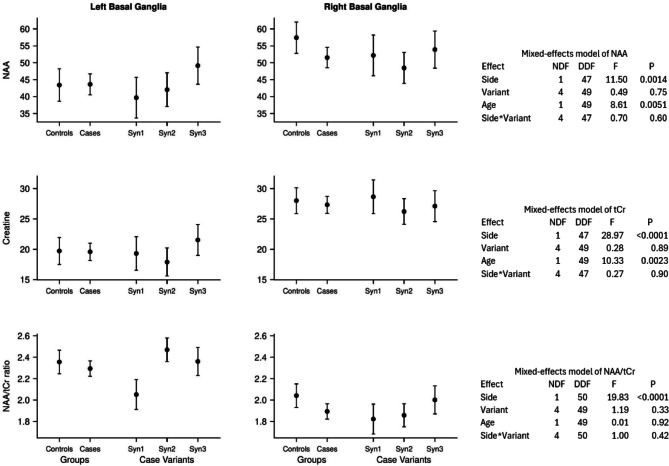
Mixed-effects repeated-measures linear models of [NAA] (N-acetylaspartate), [tCr] (total creatine) and their ratio (NAA/tCr) measured at long echo time (TE = 270 ms). For each outcome, the model tested for differences between the left and right basal ganglia (Hemisphere), between controls and case variants (Groups), and the Hemisphere-by-Group interaction. The model also controlled for age.

When measured at short TE, the mean NAA/tCr ratios of the GWI syndrome groups were consistently and substantially lower and the [tCr] in both basal ganglia were consistently and substantially higher than those of the control group; whereas, the differences of [NAA] from controls were small and inconsistent in direction (Table 2; Fig. 3). These NAA/tCr differences were statistically significant across all three syndrome groups and the pooled grouping in the left basal ganglia but not in the right basal ganglia. The increased [tCr]’s were statistically significant for all GWI syndrome groups and the pooled grouping except the syndrome 2 group. When measured at long TE, all of these differences from controls were mostly smaller and none was statistically significant (Table 2; Fig. 4). The mean myo-inositol concentrations were higher than controls for all 3 GWI syndrome groups, particularly syndrome 1, in both hemispheres, and the difference was statistically significant in the right basal ganglia (Table 3). No other metabolites or ratios differed significantly.

**Table 2:** Mean metabolite ratios and concentrations from the short-echo time (TE = 30 ms) and long echo time (TE = 270 ms) ^1^H-MRS. a.u. = arbitrary units. The values in cells are the mean metabolite ratio or concentration ± S.E.M., the percentage difference from the control group mean, and the age-adjusted 2-tailed P value from the contrast testing the difference from controls in the mixed-effects linear model. The P values in bold font remained statistically significant after controlling the overall false discovery rate of 0.05 across all contrasts and metabolites for the short-echo-time data.

Group	Left basal ganglia			Right basal ganglia		
	NAA/tCr	NAA (a.u.)	tCr (a.u.)	NAA/tCr	NAA (a.u.)	tCr (a.u.)
Short echo time (TE=30 ms)						
Controls	1.38 ± 0.03	8.07 ± 0.16	5.95 ± 0.16	1.18 ± 0.03	6.96 ± 0.16	5.91 ± 0.16
Syndrome 1	1.20 ± 0.04 -12.9% **0.0005**	8.16 ± 0.21 +1.1% 0.76	6.83 ± 0.21 +14.7% **0.0025**	1.10 ± 0.04 -7.2% 0.0832	7.31 ± 0.21 +5.0% 0.22	6.64 ± 0.21 +12.3% **0.0108**
Syndrome 2	1.28± 0.03 -6.8% **0.0227**	8.16 ± 0.16 +1.1% 0.70	6.41 ± 0.16 +7.7% 0.0487	1.13 ± 0.03 -4.2% 0.2172	7.05 ± 0.16 +1.3% 0.69	6.24 ± 0.16 +5.7% 0.1475
Syndrome 3	1.19 ± 0.03 -13.5% **0.0001**	8.48 ± 0.20 +5.0% 0.12	7.14 ± 0.20 +20.0% **<0.0001**	1.12 ± 0.03 -5.6% 0.1458	7.21 ± 0.20 +3.6% 0.33	6.47 ± 0.20 +9.5% 0.0344
Syndrome groups pooled	1.22 ± 0.02 -11.1% **<0.0001**	8.27 ± 0.11 +2.4% 0.34	6.80 ± 0.11 +14.1% **0.0001**	1.12 ± 0.02 -5.7% 0.0595	7.19 ± 0.11 +3.3% 0.25	6.45 ± 0.11 +9.1% **0.0090**
Long echo time (TE=270 ms)						
Controls	2.35 ± 0.11	43.4 ± 4.8	19.7 ± 2.2	2.04 ± 0.11	57.4 ± 4.6	28.0 ± 2.1
Syndrome 1	2.05 ± 0.14 -13.0% 0.09	39.8 ± 6.0 -8.2% 0.65	19.4 ± 2.7 -1.5% 0.94	1.82 ± 0.14 -10.8% 0.22	52.3 ± 6.0 -8.8% 0.52	28.8 ± 2.7 2.8% 0.83
Syndrome 2	2.48± 0.11 +5.1% 0.43	42.5 ± 4.8 -1.9% 0.90	18.1 ± 2.2 -8.2% 0.61	1.84 ± 0.11 -9.7% 0.19	48.5 ± 4.6 -15.4% 0.17	26.4 ± 2.1 -5.8% 0.58
Syndrome 3	2.36 ± 0.13 +0.1% 0.98	49.2 ± 5.5 +13.4% 0.43	21.6 ± 2.5 +9.4% 0.58	2.00 ± 0.13 -2.0% 0.81	53.9 ± 5.5 -6.0% 0.64	27.1 ± 2.5 -3.0% 0.80
Syndrome groups pooled	2.30 ± 0.07 -2.6% 0.64	43.9 ± 3.0 +1.1% 0.93	19.7 ± 1.4 -0.1% 0.995	1.89 ± 0.07 -7.5% 0.25	51.6 ± 3.0 -10.0% 0.31	27.4 ± 1.4 -2.0% 0.83

**Table 3:** Association of absolute myo-inositol concentrations in left and right basal ganglia with GWI. In the pooled analyses, the contrasts accounted for the correlation between the sides in a linear mixed model. The significant P values of the 2-group tests remained significant at the familywise error rate of 0.05 using Dunnett’s procedure.

Basal ganglia side	Group	Mean myo-inositol	SE	*P* values	
				2-group tests vs. control group	4-group ANOVA
Left basal ganglia					
	Controls	3.08	0.18		0.43
	Syndrome 1	3.54	0.23	0.12	
	Syndrome 2	3.14	0.18	0.81	
	Syndrome 3	3.21	0.21	0.64	
Right basal ganglia					
	Controls	2.98	0.12		0.047
	Syndrome 1	3.55	0.16	0.007	
	Syndrome 2	3.11	0.12	0.50	
	Syndrome 3	3.07	0.14	0.65	
Both sides pooled					
	Controls	3.03	0.13		0.10
	Syndrome 1	3.55	0.16	0.017	
	Syndrome 2	3.12	0.13	0.62	
	Syndrome 3	3.14	0.15	0.58	

### T_2_ relaxation times and correction factors

Calculated from the T_2_ relaxation times measured for each of the three metabolites (Table 4), the T_2_ correction factors for the metabolite concentrations measured at short TE were consistently 2–5 times smaller than for those measured at long TE (Table 5). The differences between case and control groups of the T_2_ correction factors measured at short TE were generally far smaller than the case-control differences in metabolite concentrations; whereas, those measured at long TE were generally of similar magnitude (Table 5). The relative standard errors of the T_2_ correction factors for the group means were smaller when measured by short TE than by long TE, indicating smaller variance and thus greater SNR for short TE measurements (Table 5).

**Table 4:** Calculated values of T_2_ relaxation times of NAA, tCr and tCho methyl ^1^H (mean ± SD, in ms) in basal ganglia. NAA = N-acetylaspartate, tCr = total creatine, tCho = total choline.

Metabolite	Side	Controls	Syndrome 1	Syndrome 2	Syndrome 3
NAA	Left	194 ± 29 (14)	194 ± 17 (10)	217 ± 19 (14)	206 ± 27 (11)
	Right	218 ± 19 (15)	217 ± 17 (11)	213 ± 23 (17)	211 ± 20 (11)
tCr	Left	119 ± 11 (14)	119 ± 10 (10)	127 ± 8 (14)	122 ± 13 (11)
	Right	131 ± 13 (15)	132 ± 10 (11)	131 ± 15 (17)	125 ± 8 (11)
tCho	Left	159 ± 34 (14)	157 ± 18 (10)	167 ± 19 (14)	161 ± 21 (11)
	Right	162 ± 16 (15)	162 ± 22 (10)	178 ± 20 (17)	169 ± 13 (11)

**Table 5:** Comparison of group mean T_2_ correction factors* with metabolite concentration or ratio values and their relative standard errors. SEM = standard error of the mean; syn = syndrome; long TE was 272 ms for T_2_ correction factors and 270 ms for metabolites and ratios. Short TE was 30 ms. Relative SEM is the SEM expressed as a percentage of the point estimate, which can be compared across groups. L m m. *$\rm{T_2~ correction~ Factor} = 1/(T_2 ~decay~ Factor) = 1/(e^{-TE/T_2})$.

Metabolite or ratio	Echo time	Group	Side	T_2_ correction factor		Metabolite concentration (a.u.) or ratio		
				Group mean	Case-control diff (%)	Group mean	Case-control diff (%)	Relative SEM (%)
tCr	Long	Control	Left	9.83		24.80		8.1
		Syn1		9.83	0.00	24.50	− 1.21	9.8
		Syn2		8.51	− 13.43	23.90	− 3.63	8.4
		Syn3		9.30	− 5.39	24.70	− 0.40	8.9
		Control	Right	7.98		29.15		6.9
		Syn1		7.85	− 1.63	29.15	0.00	8.2
		Syn2		7.98	0.00	27.80	− 4.63	7.2
		Syn3		8.81	10.40	27.50	− 5.66	8.0
	Short	Control	Left	1.29		5.97		2.3
		Syn1		1.29	0.00	6.72	12.56	2.5
		Syn2		1.27	− 1.55	6.30	5.53	2.2
		Syn3		1.28	− 0.78	6.80	13.90	2.2
		Control	Right	1.26		5.98		2.3
		Syn1		1.26	0.00	6.62	10.70	2.6
		Syn2		1.26	0.00	6.21	3.85	2.3
		Syn3		1.27	0.79	6.43	7.53	2.3
NAA	Long	Control	Left	4.06		50.90		8.1
		Syn1		4.06	0.00	48.20	− 5.30	10.4
		Syn2		3.50	− 13.79	41.30	− 18.86	9.9
		Syn3		3.74	− 7.88	48.10	− 5.50	9.1
		Control	Right	3.48		55.00		7.5
		Syn1		3.50	0.57	51.00	− 7.27	9.8
		Syn2		3.59	3.16	42.05	− 23.55	9.8
		Syn3		3.63	4.31	48.80	− 11.27	9.0
	Short	Control	Left	1.17		7.48		2.0
		Syn1		1.17	0.00	7.64	2.14	2.4
		Syn2		1.15	− 1.71	7.35	− 1.74	2.0
		Syn3		1.16	− 0.85	7.64	2.14	2.1
		Control	Right	1.15		7.14		2.1
		Syn1		1.15	0.00	7.36	3.15	2.4
		Syn2		1.15	0.00	6.97	− 2.31	2.2
		Syn3		1.15	0.00	7.22	1.19	2.2
NAA/tCr	Long	Control	Left	0.41		2.32		4.7
		Syn1		0.41	0.00	2.06	− 11.21	5.8
		Syn2		0.41	− 0.48	2.42	4.31	4.5
		Syn3		0.40	− 2.42	2.36	1.72	5.1
		Control	Right	0.44		2.08		5.3
		Syn1		0.45	2.06	1.83	− 12.02	6.6
		Syn2		0.45	0.90	2.00	− 3.85	5.0
		Syn3		0.41	− 8.44	1.88	− 9.62	6.4
	Short	Control	Left	0.91		1.39		2.2
		Syn1		0.91	0.00	1.21	− 12.95	2.5
		Syn2		0.91	0.00	1.28	− 7.91	2.3
		Syn3		0.91	− 0.22	1.19	− 14.39	2.5
		Control	Right	0.91		1.20		2.5
		Syn1		0.91	0.23	1.11	− 7.50	2.7
		Syn2		0.92	0.32	1.13	− 5.83	2.7
		Syn3		0.91	− 0.65	1.12	− 6.67	2.7

### Brain MRIs

In the first phase of our long-term study of this Seabees battalion in 1996, 23 of our GWI veterans (5 with syndrome variant 1, 13 with variant 2, and 5 with variant 3) and 19 of our well control veterans underwent thin section, T1-weighted MRI before and after gadolinium infusion and T2-weighted spin echo MRI of the brain on a 1.5-T magnet (Phillips Medical Systems), and no gadolinium-enhancing lesions were observed^18^. Eighteen of those 23 GWI veterans and 16 of those 19 controls remained alive and participated in the second ^1^H-MRS phase reported here.

### Screens for systemic inflammation

In the first phase we also performed 14 clinical tests in the 23 GWI veterans and 20 controls to screen for diseases that often involve systemic inflammation which can include neuroinflammation (Tables S1 and S2 in Supplementary Material 1). The case and control groups did not differ significantly on any of the tests.

## Discussion

This repeat ^1^H-MRS study of GWI case and control veterans sampled from the same Seabees Battalion found, first, that the reduction in the NAA/tCr ratio in deep gray matter identified originally in the 1997–1998 study^16^ persisted for at least 10 years up to this 2008–2009 restudy and, second, that ^1^H-MRS at higher field strength (3T) and short echo time found that the reduced NAA/tCr ratio was due to elevated [tCr] rather than reduced [NAA] as previously assumed^16^. Moreover, the [tCr] values measured at short TE were consistently elevated compared with controls across the three GWI syndrome variants and in both the right and left basal ganglia, with differences from controls being large and statistically significant in syndrome variants 1 and 3 bilaterally but paradoxically smaller and marginally significant in syndrome variant 2, the most clinically severe.

**Fig. 5:**
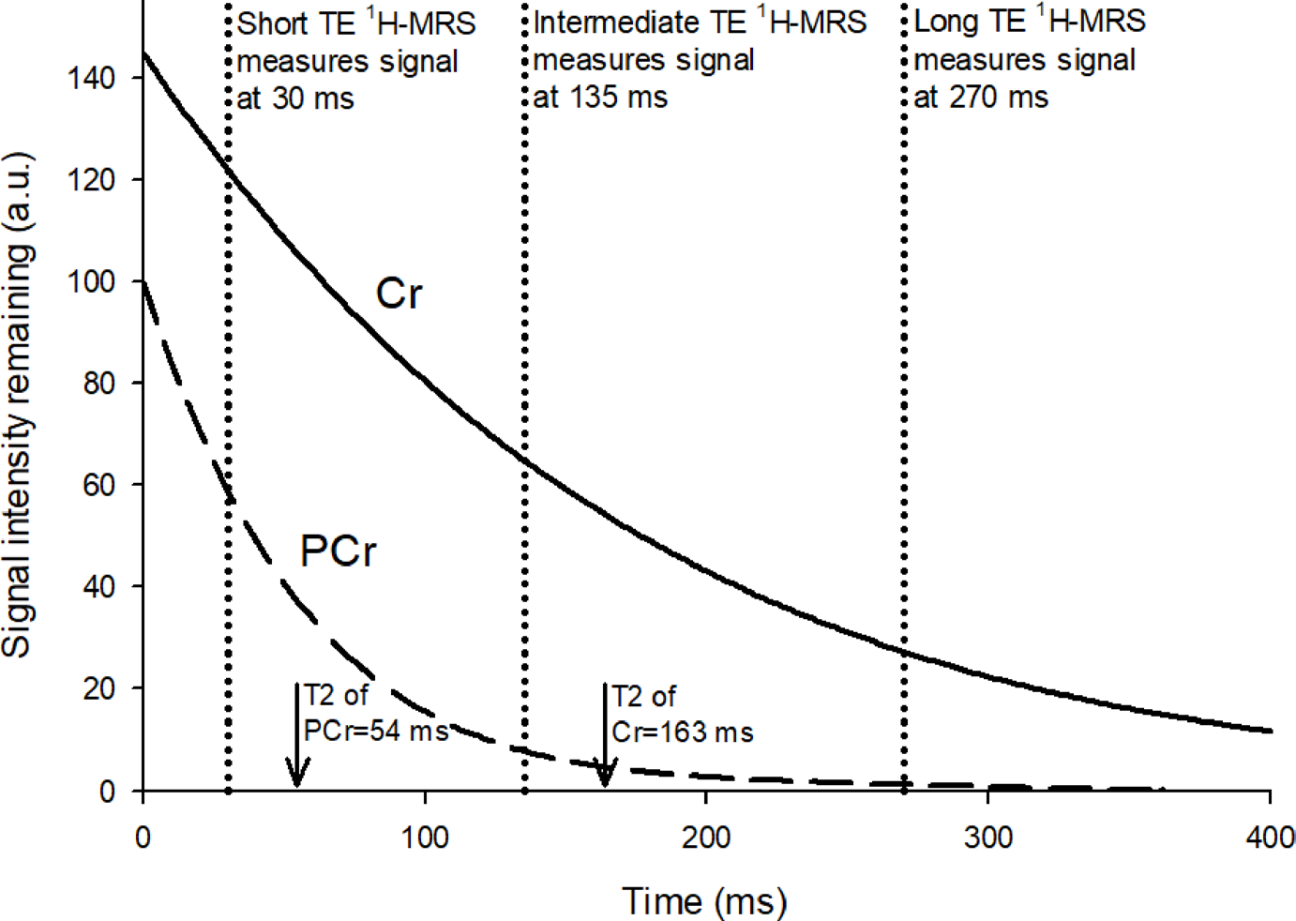
Conceptual illustration of exponential T_2_ decay curves of PCr (phosphocreatine) and Cr (creatine) following radio frequency pulse delivered at time = 0 ms. The scale of the vertical axis in arbitrary units (a.u.) reflects the fact that the concentration, and thus the signal strength of Cr at time = 0 ms before any signal decay, is 1.45 times that of PCr (this ratio at time = 0 is calculated from its known value of 1.7 at TE = 30 ms^40^. The horizontal axis is the time (ms) elapsed since the radio frequency pulse. The Cr and PCr lines show the exponential signal decay determined by their T2 decay constants which were derived as follows. The average total creatine (tCr) = 119 in the left basal ganglia of our control group (Table 4); normal brain tissue tCr is composed of approximately 59% Cr and 41% PCr; and the T_2_ decay constant of Cr is 3 times that of PCr^38^. With X denoting the T_2_ decay constant of PCr and 3X denoting that of Cr, then the T_2_ decay constant of tCr = 40.78419 · X + 59.2158 · 3X. Given, then, that the signal intensity of tCr at time = 0 is 119, the T_2_ decay constant of PCr = 54 and that of Cr = 163 (see details in Supplementary Material 2).

These findings were not detectable at long TE because of confounding by T_2_ decay. Following the magnetization from the MR magnet’s radiofrequency pulse, the signal from energy given off by the scanned object as the net magnetization relaxes back to its original orientation decays over time TE with time constant T_2_ (Fig. 5 and Supplementary Material 2). The rate of relaxation varies by chemical makeup of the scanned object; of relevance for our study, the T_2_ relaxation time of Cr is three times longer than for PCr (Fig. 5)^38^. The echo time chosen by the investigator is the point along the T_2_ decay curve where the ^1^H-MRS scan is done; short TE scans measure the signal earlier before much relaxation has occurred.

Consequently, at short echo time the case-control differences in the T_2_ correction factors were smaller than the case-control differences in metabolite concentrations and ratios (Table 5), indicating relatively little possibility of confounding. In contrast, group differences in T_2_ correction factors measured at intermediate- or long echo times (TE = 135 or 272 ms) were substantially larger and of comparable magnitude to group differences in metabolite concentrations and ratios (Table 5) and thus capable of exerting important confounding effects.

Interestingly, the signal-to-noise ratio (SNR) advantage of higher field strengths, such as 3T and 4T, is greater at short echo time, because the increased signal intensity is not as affected by T_2_ decay in signal (Fig. 5). At long echo times, however, the SNR advantage of high field strength may be substantially offset by the greater T_2_ decay, as illustrated by the greater variance of metabolite concentrations and ratios found in our study.

Of the four prior ^1^H-MRS studies of GWI, only the 2004 Menon et al. study^20^ used the more accurate short echo time at 1.5T and found significantly reduced NAA/tCr ratio in hippocampus bilaterally. The studies by Haley et al.^16^ in 2000 and Myerhoff et al.^19^ in 2001 used long echo time of 272 ms at 1.5T, and both found statistically significant reductions of the NAA/tCr ratio in the right basal ganglia and a nonsignificant trend on the left. Weiner et al.^21^ a decade later used intermediate echo time (TE = 135 ms) at 4T in a larger sample of GWI cases and controls and found no significant differences in NAA/tCr ratio in basal ganglia, pons or other deep brain structures, the authors suggesting the null finding may have been due to normalization over time. Our present negative findings at long echo time are consistent with the Weiner et al. finding of normalized [NAA/tCr] at intermediate echo time. However, our current finding of reduced [NAA/tCr] at short echo time suggests that the normalization was only partial, leaving residual reduction below the detection threshold of long echo-time ^1^H-MRS but quite visible at short echo time where the signal is less degraded by T_2_ signal decay. As illustrated in Fig. 5, at TE = 30 ms the small amount of signal decay has left large signal intensities of both Cr and PCr comprising the tCr signal detected by ^1^H-MRS; whereas, at TE = 135, and particularly at TE = 270 ms, almost all the tCr signal is from the diminished Cr with very little from PCr. Since the increase in signal strength of tCr from ATP depletion is due to both the increase in Cr and the decrease in PCr, the loss of the PCr component at higher TE degrades the change in signal strength of the tCr measurement and the decrease in tCr increases the variance of its measurement, thus obscuring the difference in signal strength between GWI cases and controls.

Understanding how our finding of elevated [tCr] helps elucidate the pathogenesis of GWI begins with the basic concept that the tCr signal increases as the cell tries to restore a depleted ATP pool by engaging the creatine kinase (CK) buffer system, which normally acts as an energy buffer in cells, particularly in tissues with high and fluctuating energy demands like the brain and muscles^39^. Potential energy is stored as PCr. When energy is needed, CK catalyzes the reversible transfer of a phosphate group from PCr to ADP to produce Cr and ATP:

$${\text{PCr}} +{\text{ADP}} \mathop{ \rightleftharpoons}\limits^\text{CK}{\text{Cr}}+{\text{ATP}}$$

As ATP is generated, Cr is added to the cytoplasm and PCr is lost. ^1^H-MRS measures [tCr] but cannot distinguish between its components, PCr and Cr. However, Cr gives off a stronger echo signal than PCr (approximately 1.7:1 in basal ganglia, measured at TE = 30 ms^40^ so that the conversion from PCr to Cr increases the signal intensity of the measured [tCr]. Thus, in the absence of rare gene mutations and nutritional creatine supplementation, increased [tCr] indicates ATP depletion, in GWI most likely from mitochondrial dysfunction^4–8^.

Inhibition of the electron transport chain during mitochondrial dysfunction reduces mitochondrial ATP synthesis, and the resulting intracellular ATP depletion activates pannexin 1 channels that paradoxically leak Ca^2+^ into the extracellular space as a distress signal^41,42^. The extracellular ATP activates P2X7 receptors on microglia that in turn activate NLRP3 inflammasomes to release cytokines IL-1β and IL-18, leading to neuroinflammation. The extracellular ATP also serves as a damage-associated molecular pattern (DAMP) that incites inflammation through NF-κβ, and impairment of the electron transport chain itself can invoke the production of reactive oxygen species and oxidative stress leading to neuroinflammation.

Our finding of elevated myo-inositol on ^1^H-MRS supports the presence of neuroinflammation in our GWI veterans in agreement with prior studies of rodent models of GWI^11,43^ and clinical studies of GWI veterans^15^. Chronic neuroinflammation can result from two basic disease mechanisms. First, it can occur in the absence of mitochondrial dysfunction from primary inflammatory diseases like brain infections (e.g., herpes encephalitis), tumors or demyelinating diseases (e.g., multiple sclerosis) and systemic inflammatory diseases (e.g., lupus) that can incite CNS inflammation that depletes ATP through its high metabolic demands. These conditions were ruled out in earlier phases of our study by negative brain MRIs with gadolinium enhancement and negative results on a panel of inflammatory blood markers in former highly fit soldiers made ill by a common environmental exposure^18^ as well as by a later clinical study^7^.

This leaves the second mechanism, dysfunction of ATP-producing mitochondria that normally maintain ATP levels in the brain. Whereas our findings showing ATP deficits cannot directly attribute it to mitochondrial dysfunction in our GWI veterans, the series of studies by Golomb et al. directly confirmed it with ^31^P-MRS demonstrating delayed PCr recovery after muscle exertion in GWI veterans^5–7^. Moreover, Deshpande et al. reported a series of studies showing that low-level sarin exposure in their rodent model of GWI caused an upward reset of intracellular calcium concentration^13,14^, and low-level sarin exposure in the Gulf War is known to have been a major cause of GWI^2,3,44^.

When cytoplasmic [Ca^2+^] remains elevated, it leads to mitochondrial Ca^2+^ overload and dysfunction^45–48^. This, in turn, reduces mitochondrial ATP production, which increases reactive oxygen species (ROS) and oxidative stress, leading ultimately to neuroinflammation^49^. The cell will attempt to restore ATP homeostasis by drawing on PCr reserves, converting PCr to Cr to generate ATP, and increasing PCr synthesis and dietary uptake to replenish those PCr reserves^39^. Furthermore, the resulting neuroinflammation increases energy demands, further driving the generation of ATP and Cr by the CK buffer system.

If, however, the elevation of intracellular [Ca^2+^] and mitochondrial dysfunction persist and become severe, PCr synthesis may be impaired, resulting in depletion of the PCr pool, drop in PCr and Cr production, and reduction in the ^1^H-MRS measure of [tCr] to normal or low levels^49,50^. This mechanism appears to explain the largest increases in [tCr] in our syndrome variants 1 and 3, which past studies have shown involve only moderately severe illness, and the smaller increases in [tCr] in our syndrome variant 2 veterans who have the most severe illness^17,51^.

The sample of Gulf War veterans studied here was drawn from the roster of the Seabees Battalion that served in the Gulf War and thus might not represent the full spectrum of the U.S. military personnel deployed to the Kuwaiti Theater of Operations in 1990–1991. Their characteristic symptoms, however, are indistinguishable from those reported from population-representative samples of the deployed^17,52^. Most importantly, they were selected for study from the battalion roster, not healthcare-seeking volunteers^17^. Fully relaxed spectra were not collected to calculate T_1_ relaxation times to assess the possibility of confounding by differences between T_1_ values of the groups. The T2 decay curves were modeled as bi-exponentials, and a single exponential decay was found to give the best result, consistent with rapid exchange averaging. Finally, intravoxel segmentation was not done to adjust for partial volumes of gray and white matter because we placed the basal ganglia single voxel mostly inside the putamen with small corners of caudate head and thalamus where there is little white matter and no CSF. Although greater atrophy in GWI veterans than controls might have altered the fractional distribution of gray and white matter slightly within the voxel, this would not have altered the Cr:PCr ratio, which has been shown to be relatively constant across gray and white brain regions^40^.

Although our study confirms that reduced NAA/tCr at long echo-time ^1^H-MRS in the 1990s in sarin-caused GWI^16,19,20^ later disappeared^21^, its continued persistence at short echo time^20^ confirms chronic pathology. Our clarification that, at least in later years, it is due to elevated [tCr] rather than reduced [NAA] supports preclinical and clinical findings of mitochondrial dysfunction, ATP deficit, oxidative stress and neuroinflammation^5,9,10,12^ as potential targets for treatment.

## Supplementary Information

Below is the link to the electronic supplementary material.

Supplementary Material 1

Supplementary Material 2

## Data Availability

The datasets generated and analyzed during the current study are not publicly available due to conditions of the assurance given to the research subjects in obtaining their informed consent, but a de-identified version of the data is available from the corresponding author upon reasonable request.

## Abbreviations

ADP: Adenosine diphosphate
ATP: Adenosine triphosphate
CDC: Centers for Disease Control and Prevention
CK: Creatine kinase
Cho: Choline
Cr: Creatine
GWI: Gulf War illness
^1^H-MRS: Proton magnetic resonance spectroscopy
NAA: N-acetylaspartate
PCr: Phosphocreatine
SNR: signal-to-noise ratio
^31^P-MRS: Phosphorus magnetic resonance spectroscopy
T: Tesla
tCho: Total choline
tCr: Total creatine
TE: Echo time
TR: Repetition time
ROS: Reactive oxygen species
