# Supplementary material for: Decadelong low basal ganglia NAA/tCr from elevated tCr supports ATP depletion from mitochondrial dysfunction and neuroinflammation in Gulf War illness
Source: Sci Rep. 2025 Nov 20;15:39741. doi: 10.1038/s41598-025-24099-0 (PMC12634682; doi:10.1038/s41598-025-24099-0)
Supplement: Supplementary file 1 — Supplementary Material 1 [file 41598_2025_24099_MOESM1_ESM.docx]

**Supplementary Material 1**

| **Test name** | **Normal range** | **Mean (SEM)**  **GWI cases**  **N = 23** | **Mean (SEM)**  **Controls**  **N = 20** | **P value*** | **Disease(s) screened/tested for** |
| --- | --- | --- | --- | --- | --- |
| Erythrocyte sedimentation rate | Men <20 mm/hr | 7.1 (1.3)† | 6.8 (1.4) | 0.86 | General inflammation (nonspecific): RA, SLE, vasculitis, infection, cancers and others. |
| High-sensitivity C-reactive protein | <3 mg/L | 2.1 (0.4) | 2.2 (0.3) | 0.84 | General inflammation (nonspecific): RA, SLE, vasculitis, infection, cancers and others. |
| IgA | 70-400 mg/dL | 233 (17) | 226 (19) | 0.80 | RA, SLE, celiac disease, chronic infection, immunodeficiency |
| IgG | 600-1,600 mg/dL | 1,091(66)† | 1,099 (62) | 0.93 | RA, SLE, chronic infections, immunodeficiencies, myelomas |
| IgM | 40-250 mg/dL | 147 (15) | 117 (16) | 0.18 | RA, infections, immunodeficiencies, Waldenström macroglobulinemia |
| C3 complement | 88–201 mg/dL | 133 (5) | 122 (5) | 0.12 | SLE, autoimmune diseases, complement deficiency |
| C4 complement | 12–38 mg/dL | 32 (2) | 33 (2) | 0.26 | SLE, hereditary angioedema, other autoimmune/inflammatory diseases |
| Acetylcholine receptor-binding antibody | <0.05 nmol/L | 0.001 (0.003) | 0.005 (0.004) | 0.45 | Myasthenia gravis |
| Creatine kinase | 22–198 U/L | 131 (12) | 141 (13) | 0.57 | Muscle injury, polymyositis, dermatomyositis, rhabdomyolysis |
| Total lymphocyte count | 1,000-4,800/μL | 2,168 (165) | 2,111 (178) | 0.81 | Immune status; high: infections, certain leukemias/lymphomas; low: viral infections, HIV, autoimmune, immunodeficiency |
| **Table S1**. Serological markers of systemic inflammatory diseases measured in the 1996 study of the sample of Gulf War-era veterans from the Seabees battalion. *By t-test. †1 high outlier excluded. | | | | | |

| **Test name** | **Normal range** | **GWI cases** | **Controls** | **P value*** | **Disease(s) screened/tested for** |
| --- | --- | --- | --- | --- | --- |
| ANA titer | Low: ≤1:40-1:80  Mod: 1:160-1:320  High: ≥1:640 | 22  1  0 | 19  0  1 | 1.00 | SLE, other connective tissue diseases, autoimmune diseases |
| Anti-dsDNA | Neg: <10 IU/mL  Pos: ≥10 IU/mL | 23  0 | 20  0 | 1.00 | SLE (highly specific); may rarely be positive in other autoimmunity |
| Rheumatoid factor | Neg:<1:40  Pos: ≥1:80 | 23  0 | 20  0 | 1.00 | RA (primary), Sjögren’s syndrome, other autoimmune diseases |
| Striated muscle antibody titer | Neg:<1:40  Pos: ≥1:80 | 22  1 | 20  0 | 1.00 | Myasthenia gravis (often with thymoma), rarely other conditions |
| **Table S2**. Serum antibody markers of systemic inflammatory diseases measured in the 1996 study of the sample of Gulf War-era veterans from the Seabees battalion. *By Fisher’s exact test. | | | | | |
