# Supplementary material for: Decadelong low basal ganglia NAA/tCr from elevated tCr supports ATP depletion from mitochondrial dysfunction and neuroinflammation in Gulf War illness
Source: Sci Rep. 2025 Nov 20;15:39741. doi: 10.1038/s41598-025-24099-0 (PMC12634682; doi:10.1038/s41598-025-24099-0)

**Supplementary Material 2**

**Steps in Calculating Values for the PCr and Cr Curves in Figure 5**

**Estimation of the [Cr]:[PCr] ratio at TE=0**

1. First, we calculated the ratio of the tissue concentrations of Cr:PCr = 1.45 at TE=0, to estimate the actual ratios before they are reduced by T2 decay. We start from the published value of the ratio 1.7:1.0 measured at TE=30 in the basal ganglia by Pouwels et al. [1] and estimate the ratio at TE=0 by the following calculations:

In our study at TE = 30 ms we measured the [tCr] in basal ganglia of controls = 6 a.u. (Table 2). From Pouwels et al. [1] we know that if the basal ganglia [PCr] = x, then [Cr] = 1.70 x and [tCr] = 2.70x = 6 a.u. and x = [PCr] = 2.22 a.u. and 1.70x = [Cr] = 3.78 a.u.

In basal ganglia at TE = 0 ms (the y-axis intercept), [Cr]_t=0_ = 3.78 a.u. x exp(30 ms/309 ms) = 3.78 a.u. x 1.10195658 = 4.165 a.u. and [PCr]_t=0_ = 2.22 a.u. x exp(30 ms/117 ms) = 2.22 a.u. x 1.292282455 = 2.869 a.u.

Thus at t = 0, [Cr]_t=0_/[PCr]_t=0_ = 4.165 a.u./2.869 a.u. = **1.45**.

Alternative calculation:

In basal ganglia at TE = 0 ms (the y-axis intercept),

[Cr]/[PCr] = 0.63/exp(-30 ms/309 ms) / 0.37/exp(-30 ms/117 ms)

= (0.63 x exp(30 ms/309 ms)) / (0.37 x exp(30ms/117 ms))

= (0.63exp(0.09708738)) / (0.37exp(0.25641))

= (0.63 x 1.10195658) / (0.37 x 1.292282455)

= 0.6942326454 / 0.47814450835

= **1.45193060524**.

These two calculations, not surprisingly, give the same value.

**Estimation of the percentage distribution of Cr and PCr in tCr**

1. Next from the tissue concentration ratio of 1.45:1.0 at TE=0, we calculated the percentage distribution for Cr and PCr in tCr as:

%Cr = 1.45193/2.45193 = **59.2158**

%PCr = 100 – 59.2158 = **40.78419**

**Estimation of the T2 decay constants of Cr and PCr**

1. Calculation of T2 decay constants of PCr and Cr:

Given (1) that 119 is the total signal intensity value for tCr by ^1^H-MRS (see Table 4 of our paper); (2) that PCr comprises 40.78% of the tCr concentration and Cr comprises 59.22% at TE=0 (from step 2 above); and (3) that from Ke et al. [2] the T2 decay constant of Cr is 3 times that of PCr, tCr is apportioned into PCr and Cr amounts to estimate the T2 decay constants by this calculation:

0. 40.78419 · X + 0. 59.2158 · 3X = 119

X = 119 / (40.78419 + 1.7764743)

X = 119 / 2.1843162

X = **54.479292** = T2 decay constant of PCr

3X = **163.43788** = T2 decay constant of Cr

**Constructing Figure 5**

1. In the table below, the X and Y values for the PCr and Cr curves were calculated as follows:

Horizontal axis: T is the time that has elapsed since the magnetization at T=0 and is plotted on the horizontal axis. Time then progresses at the rate of immediately previous T value plus the T2 constant at each step. (The T2 decay constant is the time it takes the magnetization signal to decay to 1/e (~37%) of its previous value.)

Vertical axis: The Percent of total signal remaining starts at 100 a.u. at T=0 for PCr and at 145 a.u. at T=0 for Cr and then declines at the rate of the previous PCr value times 1/e, or 1 / 2.71828. The X and Y values of Cr are derived the same way. The computation were carried out over 21 T2 values to make the most robust exponential curve fit.

1. The resulting X and Y values were entered into SigmaPlot to draw the curves, with the X axis limited to 0 to 400 ms and the Y axis from 0 to 160 a.u., and a 7^th^ order polynomial line was fit to the points.
2. Notice with the T2 decay constants of PCr = **54.479292** and Cr = **163.43788** over the relevant extent of the horizontal axis 0 to 400 ms, the PCr value declines far faster than the Cr value because of its smaller T2 constant.

**References**

1. Pouwels, P. J. *et al.* Regional age dependence of human brain metabolites from infancy to adulthood as detected by quantitative localized proton MRS. *Pediatr. Res.* **46**, 474–485, doi:10.1203/00006450-199910000-00019 (1999).

2. Ke, Y. *et al.* Biexponential transverse relaxation (T(2)) of the proton MRS creatine resonance in human brain. *Magn. Reson. Med.* **47**, 232–238, doi:10.1002/mrm.10063 (2002).

| **Calculations of values for lines in Figure 5.** | | | | |
| --- | --- | --- | --- | --- |
| **PCr** | |  | **Cr** | |
| **Time (ms)*** | **Signal remaining†** |  | **Time (ms)*** | **Signal remaining†** |
| 0 | 100 |  | 0 | 145 |
| 54.47929 | 36.78794 |  | 163.43788 | 53.34252 |
| 108.95858 | 13.53353 |  | 326.87576 | 19.62362 |
| 163.43788 | 4.97871 |  | 490.31364 | 7.21912 |
| 217.91717 | 1.83156 |  | 653.75152 | 2.65577 |
| 272.39646 | 0.67379 |  | 817.18940 | 0.97700 |
| 326.87575 | 0.24788 |  | 980.62728 | 0.35942 |
| 381.35504 | 0.09119 |  | 1144.06516 | 0.13222 |
| 435.83434 | 0.03355 |  | 1307.50304 | 0.04864 |
| 490.31363 | 0.01234 |  | 1470.94092 | 0.01789 |
| 544.79292 | 0.00454 |  | 1634.37880 | 0.00658 |
| 599.27221 | 0.00167 |  | 1797.81668 | 0.00242 |
| 653.75150 | 0.00061 |  | 1961.25456 | 0.00089 |
| 708.23080 | 0.00023 |  | 2124.69244 | 0.00033 |
| 762.71009 | 0.00008 |  | 2288.13032 | 0.00012 |
| 817.18938 | 0.00003 |  | 2451.56820 | 0.00004 |
| 871.66867 | 0.00001 |  | 2615.00608 | 0.00002 |
| 926.14796 | 0.00000 |  | 2778.44396 | 0.00001 |
| 980.62726 | 0.00000 |  | 2941.88184 | 0.00000 |
| 1035.10655 | 0.00000 |  | 3105.31972 | 0.00000 |
| 1089.58584 | 0.00000 |  | 3268.75760 | 0.00000 |
| *Time increases by the value of the T2 constant of the given metabolite at each step. | | | | |
| †Signal remaining at each step is the previous signal value times 1/*e* = 1 / 2.71828. | | | | |


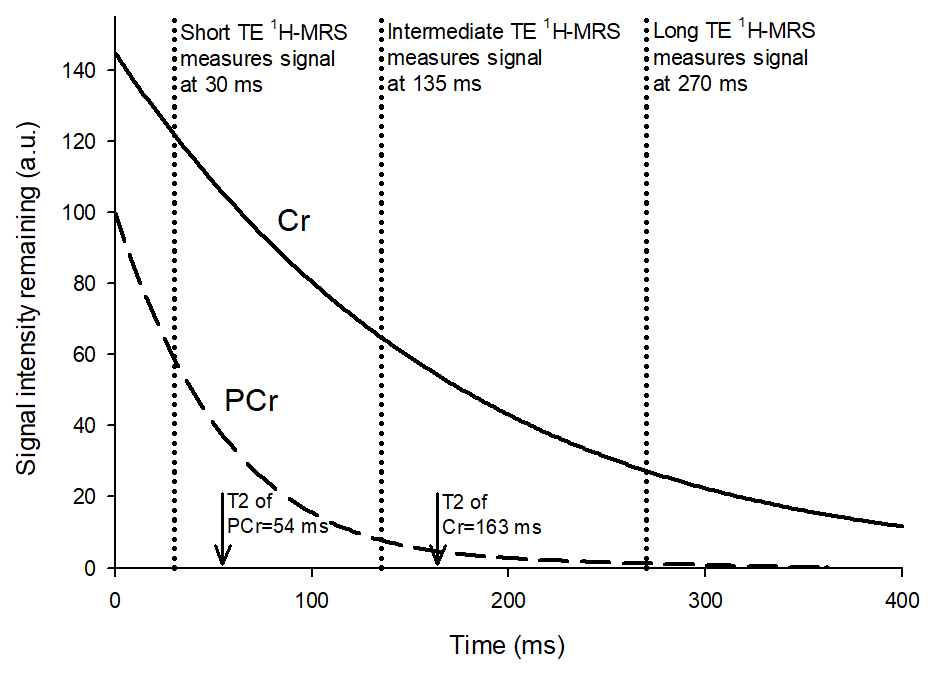

Supplement: Supplementary file 2 — Supplementary Material 2 [file 41598_2025_24099_MOESM2_ESM.docx]
